# Prevalence of tuberculosis, HIV/AIDS, and hepatitis; in a prison of Balochistan: a cross-sectional survey

**DOI:** 10.1186/s12889-019-8011-7

**Published:** 2019-12-04

**Authors:** Ahmad Wali, Dawood Khan, Nauman Safdar, Zeenat Shawani, Razia Fatima, Aashifa Yaqoob, Aurangzeb Qadir, Sultan Ahmed, Hamayun Rashid, Bashir Ahmed, Shereen Khan

**Affiliations:** 1 Provincial Tuberculosis Control Program, Health Department Government of Balochistan, Building Western Bypass Link Brewery Road, Quetta, Balochistan Pakistan; 2 National Tuberculosis Control Program, Ministry of NHSR&C, Islamabad, Pakistan; 3 Provincial AIDS Control Program, Quetta, Balochistan Pakistan; 4 Social and Health Inequalities Network Pakistan, Quetta, Pakistan; 5 University of Bergen, Bergen, Norway; 6 Mercy Corps, Quetta, Pakistan; 7 Faculty of Bolan Medical University, Quetta, Pakistan

**Keywords:** Prison, HIV, Hepatitis, Tuberculosis, Screening, Prevalence, CAD4TB, Active-case-finding

## Abstract

**Background:**

Human Immunodeficiency Virus (HIV), Hepatitis, and Tuberculosis (TB) are three primary communicable infections have the likely potential to cause severe morbidity in prison settings. The prison has the most favorable environment for the transmission of infections. We conducted this survey to determine the prevalence and feasibility of rapid diagnostic tests in an active screening of these infectious diseases in prison.

**Methods:**

This cross-sectional survey conducted in central Jail Gaddani, one of the largest prisons in the Balochistan province of Pakistan. All prisoners, jail staffs, and staff’s family members participated. Informed consent obtained from each participant before the screening. Van equipped with digital X-ray linked with Computer-Aided Detection for TB (CAD4TB) software used for testing. Sputum samples tested on Xpert for MTB/RIF assay and blood specimens collected for HIV and hepatitis serology. Diagnosed TB patients enrolled for treatment at Basic Management Unit (BMU), reactive on hepatitis Rapid Diagnostic Tools (RDTs) were referred for further testing and management, while HIV reactive referred to Anti Retro Viral (ARV) center for Anti Retro Viral Treatment (ART).

**Results:**

A total of 567 participants offered screening, 63% (356) prisoners, 23% (129) staff’s family members, and 14% (82) jail staffs. Among tested 10.3% (58/562) were hepatitis seropositive (Hepatitis-C 41 [7.29%] Hepatitis-B, 16 [2.84%] Hepatitis B&C both, 01 [0.17%]). In reactive participants, 49 were prisoners, 08 were jail staffs, and 01 was the staff’s family member. HIV seropositive was 4% (24/566), and all were prisoners. Almost 99% (565/567) screened by digital X-ray, 172 (30%) were with abnormal CAD4TB suggestion (score > 50), out of them sputum of 26% (148) tested on Xpert, and 2% (03) found *Mycobacterium tuberculosis* Positive (MTB+). A total of five TB patients were detected; out of two were diagnosed clinically. Co-morbidities observed in 15 patients, (01 TB/HIV co-infected, 12 HIV/HCV, 01 HIV/HBV, and 01 HBV/HCV).

**Conclusion:**

The high frequency of infectious diseases in prison is alarming. For limiting the transmission of infections among prison and community, immediate steps are needed to be taken for improvement of prisons condition by application of recommended screening protocols at the time of the first entry of prisoners in prisons.

## Background

Prisons act as a reservoir for infectious diseases and inflating the disease into the community through staffs, visitors, and releases of inadequately treated prisoners [1–3]. According to World Prison Brief, in 2015; more than 10.35 million prisoners either as pre-trial/remand /convicted or sentenced are under in penal institutions in 223 prisons. During the same period; about 80,169 persons (pre-trial/remand/ convicted/sentenced) were in prisons in Pakistan, which was 171% occupancy level of actual (40705) official capacity of the prison system [4].

.Prisons reported with inadequate medical care services, overcrowding and poor hygienic conditions in general [5]. The injurious/high-risk activities i.e., injecting drugs, alcoholism, unprotected and multiple-partner sexual activity, barbering, and smoking, are associated with overcrowded prisons. Which inversely contribute to the poor health of the individual and higher risk of infection transmission and development of disease in later ages [1, 6–10]. Globally, the majority of the prisoners belong to age group of15–44 years and of low socioeconomic status and having inadequate education. They are also frequently subjected to avoidable health risks: through lack of access to screening, immunization, and active case-finding programs [10–12]. Screening of prisoners and jails staff and staff’s family members could provide a high-yield opportunity for early disease detection and timely treatment to control infection in prisons and the general population [3].

.Pakistan is a double disease burden country; facing the challenge of the common epidemic and endemic infectious diseases [13]. Globally; Pakistan is the 5th highest TB burden and 2nd highest viremic infection country [14, 15]. In 2017; a total 368,897 TB cases notified against the estimated burden of 525,000 [16]. HIV and Acquired Immunodeficiency Deficiency Syndrome (AIDS) epidemic among the general population in Pakistan is less than 0.1%. There is a shift from “Low prevalence in key population’ to a more concentrated epidemic in Pakistan [10]. In 2016; an estimated 130,000 (120,000-150,000) people living with HIV in Pakistan [17]. In 2017; more than 10 million people infected by Hepatitis in Pakistan [18]. Hepatitis B&C prevalence are 2.5 and 4.8% respectively, with co-infection combined rate of 7.6% in the general population in Pakistan [15].

.Prisons in Balochistan like other parts of the country are also overcrowded and might be one of the key contributing factors to poor prison conditions and health status [12, 19, 20]. Preventive programs like TB, AIDS, and hepatitis control programs don’t provide permanent screening and management setups within the prisons.

Globally in prisons, high prevalence, and wide transmission of HIV, Hepatitis-B Virus (HBV), Hepatitis-C Virus (HCV) and TB is witnessed than the general population. In addition to the challenges faced in a prison setting, there are opportunities for early detection and management of these highly infectious diseases [4, 10–12, 18, 21, 22]. In south Asia, prisons recognized as hubs of infectious diseases. Statistics on the burden of HIV, HBV, HCV, and TB in prisons have not well documented, and therefore, information comes mainly from studies conducted in developed countries [20, 23]. Research studies in Pakistan have focused on disease detection, treatment, community linkages, health education, and prevention for prison inmates [3, 5, 20, 22, 24]. Limited shreds of evidence founded for research, survey or screening through active case finding and utilization of advance screening, and diagnostic tools with high sensitivity and specificity for the assessment of the prevalence of three major infectious diseases collectively. This is likely to underestimate the overall magnitude of the problem; because a large number of prisons and the prison population are there.

This study aimed to assess the prevalence of three infectious diseases HIV, Hepatitis B-C, and TB among prisoners, jail staffs, and staff’s family members.

## Methods

It was a cross-sectional study. Permission obtained from the Inspector General (IG) Prisons through provincial home department. During camp, informed written consent included questionnaires pursued from each of the participants. Parents in case of children (signature, writing name, and thumb impression) before administration of screening to collect personal and socio-demographic data.

### Study site

This study conducted in central Jail Gaddani, district Lasbela of Balochistan province in Pakistan.

### Setting

#### General setting

Balochistan is one of the four provinces of Pakistan situated on the southwest part of the country. It is the largest province and covers an area of 347,190 km^2^, constituting approximately 44% of the total land area of Pakistan and comprises of 33 districts [25]. In the year 2017, the population estimated at 10.2million, which scattered across the problematic terrains.

Prison department serves under the provincial home department. There are eleven ‘11’ prisons/jails in Balochistan. Of these, four are the larger “Central” and seven are the relatively smaller “District” jails. Women have no separate jails of their own, and they lodged in a section of men’s prison along with their children. Prisons in Balochistan are overcrowded than the actual number of facilities available for intimates, which is one of the key contributing factors to poor prison conditions health status [8, 21, 22]. Provision of health services in prisons are under the provincial department of health, includes the provision, management, and development of human resource and regulatory functions. Insufficient and shortage of health staffs in prisons always remain a challenge in Balochistan. Preventive programs such as TB, HIV/AIDS, and hepatitis control programs don’t provide permanent screening and management setups within the prisons. Patients presented with symptoms of TB within the hospitals of Jail referred to District Head Quarter (DHQ) hospitals and/or specialized hospital for investigation and diagnoses. Diagnosed patients managed from the facility to follow up visits when needed.

#### Specific setting

Central Jail Gaddani is one out of four larger jails in Balochistan prison department. Gaddani is a coastal village situated in tehsil Hub of district Lasbela at the southern part of province Balochistan. This site is both Jail and prison (Jails usually designed to hold inmates awaiting trial or serving a short sentence and prisons designed to hold intimates for long and individuals convicted of more serious crimes) [26]. It serves as the principal imprisonment venue for adult males with illicit drug allegations, as well as for men accused of many other crimes. Central Jail Gaddani is mainly catering the inmates from the districts of Makran and Kalat divisions of Balochistan province. A survey conducted in 2016, indicated a high prevalence of HIV,(16.6% in PWIDs, 1.8% in TG-SW and 1.9% in MSM) in Turbat district Kech and (6.0% in PWIDs and 3.7% in MSM) in central Jail Gaddani [9, 10], with underlying unprotected sexual, smoking, alcoholism and injecting behaviors, determinants of the TB/HIV co-infection, HBV and HCV infections in prison environment. Rural health center (RHC) Gaddani is the nearest BMU (5 km), while Jam Ghulam Qadir (JGQ) civil hospital Hub (25 km) is the nearest Xpert and TB/HIV sentinel site with central Jail Gaddani.

### Study population

#### Inclusion criteria

All prisoners, jail staffs, and staff’s family members with the informed consent of central jail Gaddani during the study period included.

#### Exclusion criteria

All prisoners, jail staffs, and staff’s family members who refused to participate, were not present during screening, seriously ill, known HIV, Hepatitis and tuberculosis patients, excluded from the study.

### Sampling methods and sample size

Central Jail Gaddani consists of 20 barracks of different sizes, in addition to a hospital barrack; prisoner population ranges from 2 to 20 per barrack. We sampled all prisoners, staffs of the Jail, and their family members in the staff’s residence colony to obtain more precise estimates of infectious diseases.

### Data collection and validation

Data was collected using a structured data collection form ‘An additional file shows this in more detail (see Additional file 1)’ by the trained study team. For each prisoner participant included in the study; we determined the status of imprisonment, occupation, drug use, rural/urban residence. The prison ID numbers along with the prisoner’s name, sex, and age, which used for tracking the diagnosed patients’ on positive/reactive results of screening tests. The study team included trained technical staff for Xpert testing, digital X-ray processing in mobile vans, laboratory techniques (RDTs for HIV and Hepatitis), and sputum sample collection for Xpert testing. A medical officer, chest specialist, and child specialist were available for clinical evaluations and diagnosis. All Jail health care staff and study team were trained/oriented in ethical consideration of privacy and confidentiality.

### Outcome definition

For the study purposes, we defined TB patient if any participant detected MTB by Xpert testing, or clinically diagnosed by study physicians after an abnormal finding on CAD4TB digital X-ray. HIV patients who confirmed on three serial testing RDTs. Hepatitis infected patients who ruled on one serological assay testing reactive result on RDT.

### Screening methods

#### Tuberculosis (TB)

##### Screening through digital X-ray mobile van linked with CAD4TB software

All participants screened through a digital X-ray machine connected with CAD4TB, which can immediately analyze digital images made by digital X-ray machines installed in a mobile van. Digital X-rays can efficiently make large numbers of chest radiographs at a low cost. CAD follows the processing steps, 1- Lung shape analysis, 2- Clavicle detection, 3- Texture analysis. Texture within the lung fields and the shape of the extracted lung fields compared with a training database obtained from thousands of training images. Based on this analysis, a grade for the image computed. Based on the category and expected prevalence in the population, the probability that the picture contains signs of TB calculated. The image sent automatically to a separate computer on which the CAD4TB software installed, the program performs the quality check and the image analysis steps, and the result stored on disk. The CAD4TB software gives a probability percentage of normal vs. abnormal consistent with TB [27].

##### .Sputum collection, transportation, and testing through Xpert for MTB/RIF assay

Presumptive TB case (PTC) identified by CAD4TB digital X-ray machine and as per National TB guideline, were tested for MTB/RIF assay by Xpert machine. Sputum samples obtained through front-loading technique (same-day diagnosis) [1]. Samples were preserved in specimen transporting boxes and transported to Xpert site at JGQ hospital hub for processing on the same day.

Diagnosed TB patients were enrolled in treatment for 6/8 months under national TB guideline, with provision of anti TB treatment (ATT) drugs and linked with TB BMU for follow up and completion of treatment.

### HIV/AIDS

Participants with informed consents were screened by rapid diagnostic kits (RDT) according to national HIV testing and counseling (HTC) strategy of three serial testing (Test 1 = Alere Determine HIV-1/2 Ag/Ab Combo (Sensitivity > 99%), Test 2 = Uni-Gold HIV (Specificity > 99%) Test 3 = SD Bioline HIV-1/2 3.0 (Sensitivity > 99%, Specificity > 98%).The CD-4 test for deciding to initiate Anti-Retroviral Therapy (ART) done for participants who found positive on all three following tests (T-1, T-2 & T-3) [28–30]. ART recommended through ARV centers of the AIDS control program.

### Screening for HBV and HCV

Participants with informed consents screened for HBV and HCV by RDTs and one assay serological testing strategy. Non-reactive participants vaccinated for Hepatitis-B as per the national hepatitis vaccination schedule (1st dose on the spot, 2nd dose after 1 month, and booster dose after 6 months). Participants reactive on initial RDT results were recommended to refer for further HBV DNA and HCV RNA NAT testing to confirm the diagnosis and management at concern point [29, 31, 32].

### Analysis and statistics

Data were double entered, validated, and analyzed using EpiData software (version 3.1 for entry and 2.2.2.183 for review, EpiData Association, Odense, Denmark). Initially, descriptive statistics were used to describe the study population and diagnosed TB, AIDS, HBV, and HCV patients.

## Results

**Fig. 1:**
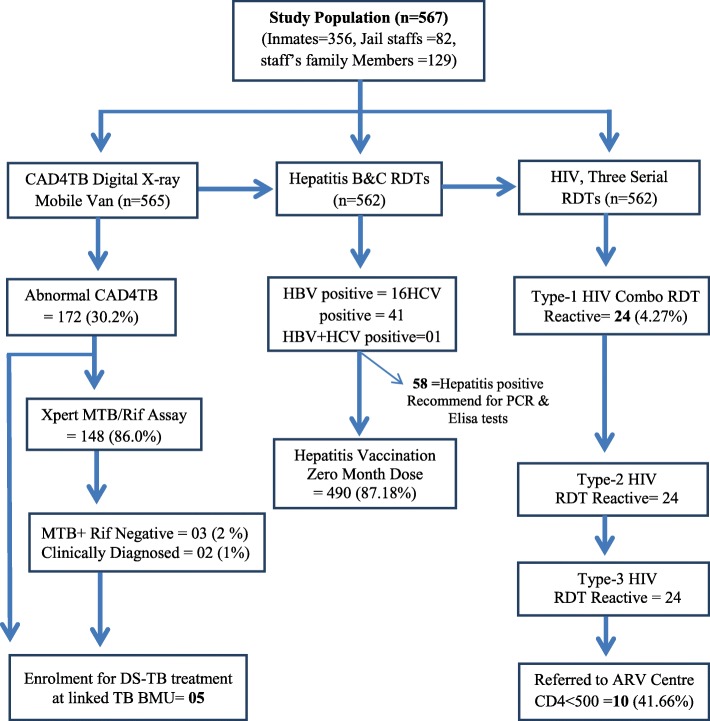
Flow diagram of systematic screening for HIV, Hepatitis and TB in central Jail Gaddani Balochistan

A total of 567 participants offered screening, 63% (356) prisoners, 23% (129) jail staff’s family members, 14% (82) jail staffs, and less than 1% refused the screening Fig. 1. Almost all participants were screened initially by questionnaire. The median age of study participants was 30 years (IQR: 21–37) [30 years (IQR: 26 ± 37) in the prisoners, 32 years (IQR: 24 ± 39) in the staff’s family members and 33 years (IQR: 29 ± 40) among jail staffs]. Among enrolled majority, participants were male (85%). A total of 58 participants detected as HCV and HBV reactive on RDT, 24HIV responsive on three series testing, and 05 TB patients. The essential socio-demographic characteristics summarized in Table 1.

**Table 1:** Socio-demographic characteristics of participants screened for HIV, Hepatitis and TB in Central Jail Gaddani, Balochistan

	Jail Inmates	Jail staffs	Staff’s family	Total
	*n* = 356 (%)	*n* = 82 (%)	*n* = 129 (%)	*N* = 567(%)
Age in years				
00–14	0 (0.0)	0 (0.0)	71 (55.0)	71 (12.7)
15–29	158 (44.4)	22 (26.8)	29 (22.5)	209 (36.9)
30–44	150 (42.1)	49 (59.7)	16 (12.4)	215 (37.8)
45–59	29 (8.1)	11 (13.4)	6 (4.7)	46 (8.1)
60 & above	19 (5.3)	0 (0.0)	7 (5.4)	26 (4.6)
Sex				
Male	351 (98.6)	76 (92.7)	57 (44.2)	484 (85.4)
Female	5 (1.4)	6 (7.5)	72 (55.8)	83 (14.6)
Residence				
Rural	78 (21.9)	12 (20.7)	20 (15.5)	115 (20.3)
Urban	278 (78.1)	65 (79..3)	109 (84.5)	452 (79.7)

*HIV* Human immunodeficiency virus

### TB diagnostic and CAD4TB probability status

Among consented; 99.6% (565/567) participants screened by digital X-ray linked with CAD4TB software for TB, out of 30% (172) were found with an abnormal suggestion (score > 50) and out of 26% (148) were tested on Xpert MTB/RIF assay. 2% 903) of the tested were found MTB detected (Fig. 1). A total of 1% (05) participants diagnosed as TB patients (02 prisoners, 02 staff’s family members, and 01 jail staff). Two children were diagnosed clinically from the staff’s family members. The probability of TB diagnosis with CAD4TB suggestion of abnormality (Score > 50) was one time more than in the opinion of normality (Score < 50), (RR = 1.03 (95% CI: 1.00–1.06) *p* = 0007).

### HIV serological testing status

The HIV seropositivity was 4% (24/562), on three series testing, 24 diagnosed as AIDS patients, and all were prisoners. The detail of three series HIV testing and CD4 count summarized in Table 2. Among 24 HIV reactive participants, 01 dually infected with TB and 13 with Hepatitis (01 HBV, 12 HCV).

**Table 2:** Screening & diagnostic tests results for HIV, Hepatitis and TB in central Jail Gaddani, Balochistan

Infection & Tools	Jail Inmates	Jail staffs	Staff’s family	Total
	*n* = 356 (%)	*n* = 82 (%)	*n* = 129 (%)	*N* = 567(%)
Tuberculosis (TB)				
CAD4TB digital X-ray	356 (100)	82 (100)	127 (98.4)	565 (99.6)
Abnormal (Score > 50)	109 (30.6)	26 (31.7)	37 (28.7)	172 (30.4)
X-pert MTB/Rif Assay	120 (33.7)	12 (14.6)	16 (12.4)	148 (26.1)
MTB+ Rif-^^	02 (0.6)	01 (1.2)	00 (0.0)	03 (0.5)
Clinically diagnosed	00 (0.0)	00 (0.0)	02 (100)	02 (0.4)
HIV Three Serial RDTs	356 (100)	81 (98.8)	125 (96.9)	562 (99.1)
Type-1 reactive	24 (6.7)	00 (0.0)	00 (0.0)	24 (4.3)
Type-2 reactive	24 (6.7)	00 (0.0)	00 (0.0)	24 (4.3)
Type-3 reactive	24 (6.7)	00 (0.0)	00 (0.0)	24 (4.3)
CD4 Count^a^	19 (79.1)	00 (0.0)	00 (0.0)	19 (79.1)
< 500	10 (2.8)	00 (0.0)	00 (0.0)	10 (1.77)
Hepatitis Positive RDT	356 (100)	82 (100)	122 (94.6)	560 (98.8)
Hepatitis B positive	15 (4.2)	01 (1.2)	00 (0.0)	16 (2.9)
Hepatitis C positive	37 (10.4)	03 (3.7)	01 (0.8)	41 (7.3)
HBV+ HCV positive	01 (0.3)	00 (0.0)	00 (0.0)	01 (0.2)

^a^CD4 Count done for reactive on 3 series HIV RDTs; *CAD4TB* Computer aided detection for TB, *RDTs* Rapid diagnostic tests, *MTB+/Rif- Mycobacterium tuberculosis* Detected/Rifampicin resistant not detected, *HIV* Human immunodeficiency virus, *HBV* Hepatitis B Virus, *HCV* Hepatitis C Virus

### Hepatitis serological testing status

Overall 10% (58) participants were found hepatitis seropositive; 7.29% (41) were HCV, 2.84% (16) were HBV, and 0.17% (01) was both HBV and HCV. Among hepatitis seropositives 49 were prisoners, 08 were jail staffs and 01wasstaff’s family member.

### Co-morbidities status

**Fig. 2:**
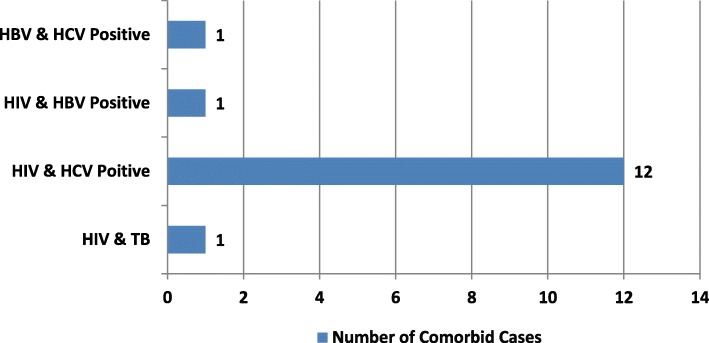
Co-morbidities among screened population for HIV, hepatitis and TB in central jail Gaddani Balochistan. (*n* = 562)

We found co-morbidities among 15 identified infected participants, 01was TB/HIV co-infected, 12 HCV/HIV, 01 HBV/HIV, and 01 HBV/HCV (Fig. 2). Prisoners were more likely to have multiple infections (100%) than other participants.

Furthermore; high uptake of infectious diseases detection through new screening tools with minimal utilization of the program resources, human resource and time with a brief orientation to health staffs to utilize these tools for prevention may be particularly feasible, reliable and highly acceptable in prison settings.

## Discussion

Our study determines the high frequency of infectious diseases (HIV, HBV, HCV, and TB) at Central Jail Gaddani, one of the largest prisons in Balochistan. The overall frequency rate of Hepatitis was 9.6%, HIV 4.2%, and TB 1%. We found the frequency of HIV, Hepatitis, and TB higher than the general population in Pakistan. This result harmonizes with studies conducted in other countries where the frequencies of infectious diseases prevalence among prison inmates were also higher compared to the general population [2, 10, 12, 14, 17]. The numbers of HIV has found significantly high as compared to the jails of other provinces of the country, which was reported 1–3% on average [33]. We also noted the co-morbidities among the infected participants with higher in HIV-infected individuals. The highest observed of HCV with HIV, because the spread routes of both are common, and so are expected to co-infect the same individuals.

Detection of significant infectious diseases patient’s in jail staffs and their family members along with intimates evident the consequences of infectious diseases in prisons which are not restricted to within the prisons. These contagious infections from inmates possibly will spread through visitors, prison staff, and released inmates into the community, thereby make vulnerable the efforts done in the general population for the control of infectious diseases.

In this study, active case finding with newly recommended sensitive and specific screening tools for infectious diseases detected 58 cases of Hepatitis, 24 cases of HIV, and 05 cases of TB at Central Jail Gaddani. Screening by digital X-ray linked with CAD4TB evident the time and actively diagnosis of TB patients with its suggestion (Score < 50) of abnormality, which was one time more than the normal. The outcome of this study, therefore, highlights the significance of active case finding of infectious diseases in prisons. The choice of the best latest recommended screening tools and diagnostic algorithms will remain an essential deliberation in the control of contagious diseases in prisons.

There were several strengths of the study. First, the study had a representative sample size and screened all the prisoners, jail staffs, and staff’s family members with a high response rate. Second, our action plan provided better logistic measures to systematically use latest screening tools and diagnostic algorithms with high sensitivity and specificity for all three major infectious diseases (digital X-ray linked with CAD4TB software, sputum testing on Xpert, WHO recommended three series RDTs for HIV, and Hepatitis, and CD4 count for HIV reactive patients). Each of these tests considered separately have their limitations, and taken together; they provide us with a higher assured estimate that should be regarded with caution. All the procedures support us for robust convinced estimates. Third, we also used an arrangement of clinical criteria with acid-fast bacilli (AFB) and radiological screening to document non-culture proven TB. Fourth, the study used the resources of the existing program in collaboration with other programs. Fifth, all participants found eligible for Hepatitis-B, vaccination was vaccinated and linked with current district hepatitis vaccination program for completion of the remaining doses as per schedule.

Limitations of this study include our incapacity to assess incidence due to cross-sectional study design, research of a particular jail due to budget constraints, and we had insufficient numbers of actions to examine risk factors for infectious diseases. Finances limited the extent of our workup and the number of jails inclusion for infections that we could diagnose.

Based on the study results, we presumed the possible reasons for the remarkably high frequency of infectious diseases in prisons, could be explained by; One, the conditions of the deprived detention. Second, inadequate health care services because the prison health system works in complete isolation from the overall healthcare system. Third, most often underfunded prison’s health care system. Fourth, the lack of screening facilities from infectious diseases controls programs: fifth, lack of awareness and counseling of the detected patients with contagious diseases. Sixth, the newly admitted prisoners, do not screen for infectious disease at the time of the first admission.

There are several program implications and considerable efforts that can be done to address the challenge of the high prevalence of infectious diseases in the prisons, which outlined in the study discussion. First, it will be necessary to take immediate actions for the betterment of prisons conditions and decrease overcrowding. Formulation of strategies based on the recommendations of different commissions and committees to reform the prison system in Pakistan is urgently needed [25]. Second, the prison healthcare system needs for increased funding and should recognize as an integral part of the public health sector. It shall advance from its present reactive “sick call” model into a proactive system that emphasizes early disease detection and treatment, health promotion, and disease prevention. Third, prison healthcare services should directly work with national infectious disease control programs to make sure that prison inmates are regularly screened for infectious diseases. Those detected positive should be put on treatment without delay by linking them with the nearest infectious disease controlling health care facility. Finally, all prisoners must be screened for infectious diseases prior to admittance into the prison/jails, and any prisoner with an infectious disease should be properly tracked up after release from the prison/jail to ensure completion of a particular treatment and/or vaccination [34].

## Conclusion

The study advocates high frequency of infectious diseases among prisoners in prisons of Balochistan, Pakistan. To limit the transmission of infections, it is necessary to take steps for improvement of prisons conditions and application of recommended screening protocols at the time of the first entry of prisoners in the prisons.

## Supplementary information

**Additional file 1.** Data Collection Form being utilized during data collection process.

## Data Availability

The datasets generated and/or analyzed during the current study are not publicly available due to maintaining the confidentiality of participants keeping in view the ethical consideration for stigmatized infectious diseases i.e. TB and HIV but are available from the corresponding author on reasonable request.

## Abbreviations

AFB: Acid-fast bacilli
AIDS: Acquired Immunodeficiency Deficiency Syndrome
ART: Anti Retro Viral Treatment
ARV: Anti Retro Viral
ATT: Anti TB treatment
BMU: Basic Management Unit
CAD4TB: Computer-Aided Detection for TB
HBV: Hepatitis-B Virus
HCV: Hepatitis-C Virus
HIV: Human Immunodeficiency Virus
IG: Inspector General
JGQ: Jam Ghulam Qadir
MTB+: *Mycobacterium tuberculosis* Positive
RDTs: Rapid Diagnostic Tools
RHC: Rural health center
TB: Tuberculosis
