# Supplementary material for: Prevalence of tuberculosis, HIV/AIDS, and hepatitis; in a prison of Balochistan: a cross-sectional survey
Source: BMC Public Health. 2019 Dec 4;19:1631. doi: 10.1186/s12889-019-8011-7 (PMC6894348; doi:10.1186/s12889-019-8011-7)
Supplement: Supplementary file 1 — Additional file 1. Data Collection Form being utilized during data collection process. [file 12889_2019_8011_MOESM1_ESM.docx]

**Consent and Data Collection Form**

Consent and socio-demographic profile, screening tests/results and diagnosis during screening camp at central Jail Gaddani. (One form will be filled for each enrolled study participant)

| **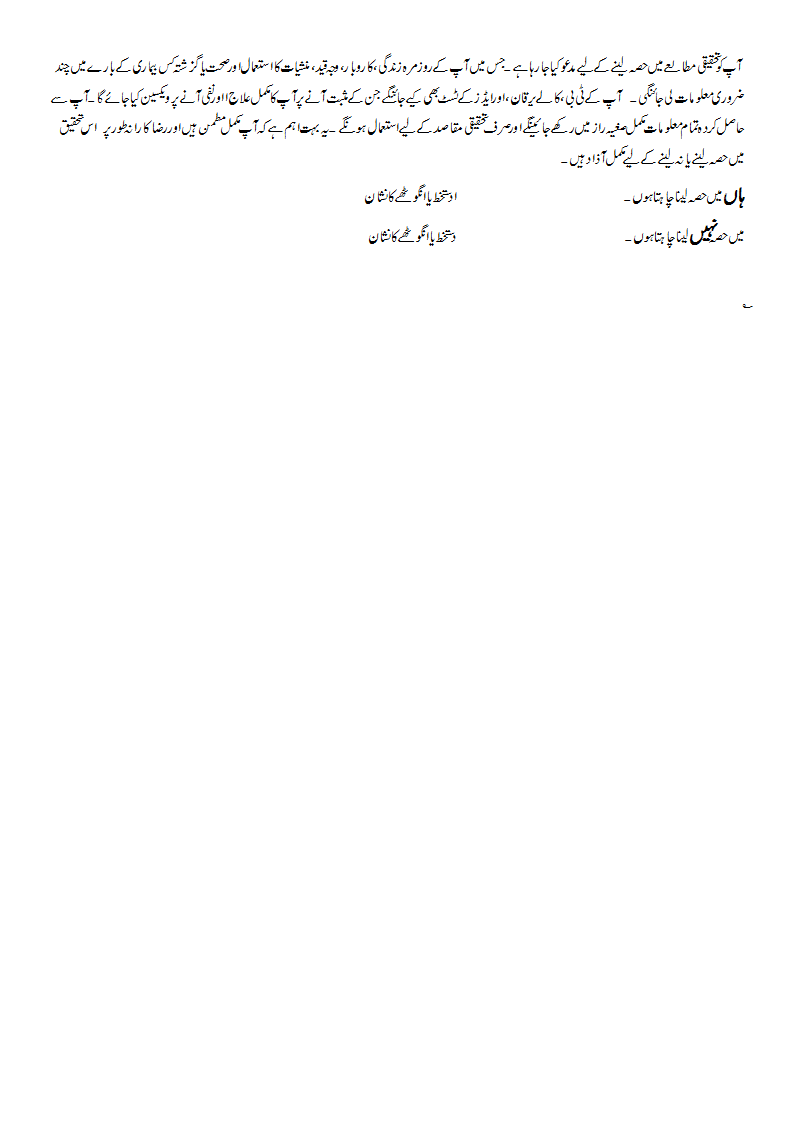** | | |
| --- | --- | --- |
| **Name:** ___ **Father/Husband Name:** _______________________________  **Age:**________________ **Date of screening:** Day________________ Month_______________ Year_______________ | | |
| **Serial No:** | **Enrolment Category:**   1. Prisoner 2. Staff 3. Staff’s Family Member | **ID Number:**   1. Prison 2. Staff 3. Staff’s Family |
| **Sex:**   1. Male 2. Female 3. Transgender | **Permanent Address:** (mention name of village, city & district) | **Residence:**   1. Rural 2. Urban |
| **Occupation:**   1. Employed 2. Businessman 3. Farmer 4. Driver 5. Other   (___________________) | **Risk Factors for TB:**   1. Contact of TB patient 2. HCP in TB setup 3. HIV positive 4. Smoker 5. Past TB patient 6. Cool Mine Worker | **Risk Factors for HIV & Hepatitis:**   1. PWID 2. Sexual History 3. Other Drug User 4. Blood Transfusion 5. Clean blade at barber |
| **CAD4TB X-ray suggestion:**  1. Normal  2. Abnormal  8. Error | **X-pert result:**  1. MTB Negative  2. MTB Rif Positive  3. MTB Rif Negative  4. Not done | **TB Diagnosed:**  1. Pulmonary  2. Extra Pulmonary  3. Not Diagnosed |
| **HIV RDT: Reactive N-Reactive**  1. Type-1 Comb  2. Type-2  3. Type-3 | **HIV RDT+, CD4 count:**   1. >500 2. <500 3. Not done | **AIDS Diagnosed:**   1. Yes 2. No |
| **Hepatitis RDT result:**  1. Reactive  2. Negative  8. Error | **Remarks:** mention any additional and relevant details like. | |
